# Chronic Recurrent Septic Peritonitis in a Diabetic Dog Caused by *Staphylococcus pseudintermedius* With Splendore–Hoeppli Reaction (Botryomycosis‐Like/Bacterial Pseudomycetoma)

**DOI:** 10.1002/vms3.71324

**Published:** 2026-11-07

**Authors:** Diamond Taylor, Kyle L. Granger, Anne Cohen

**Affiliations:** ^1^ Small Animal Internal Medicine Service MedVet Chicago Chicago Illinois USA; ^2^ Small Animal Emergency & Critical Care Service Thrive Pet Healthcare Specialists–Hoffman Estates Hoffman Estates Illinois USA; ^3^ Small Animal Emergency & Critical Care Service MedVet Chicago Chicago Illinois USA

**Keywords:** chronic infection, diabetes mellitus, meropenem, peritoneal cavity, peritoneal dialysis, peritoneal fluid, peritonitis, septic peritonitis, *Staphylococcus pseudintermedius*, surgery

## Abstract

A 6‐year‐old neutered male Miniature Pinscher with poorly regulated diabetes mellitus was evaluated for recurrent abdominal effusion, lethargy and inappetence secondary to chronic septic peritonitis. Across four discrete clinical episodes, *Staphylococcus pseudintermedius* was repeatedly isolated in pure culture from peritoneal fluid and omental tissue, with consistent antimicrobial susceptibility profiles across submissions, a pattern suggestive of strain persistence rather than serial reinfection. Histopathology of omental biopsies demonstrated severe, chronic pyogranulomatous peritonitis with prominent Splendore–Hoeppli material surrounding bacterial colonies, consistent with bacterial pseudomycetoma. Despite two exploratory laparotomies with extensive abdominal lavage, closed‐suction peritoneal drainage, broad‐spectrum systemic antimicrobial therapy, and a subsequent salvage protocol of combined intravenous and intraperitoneal meropenem administered via an ultrasound‐guided peritoneal catheter, infection ultimately recurred and the dog was humanely euthanized. The case is consistent with biofilm‐associated chronic infection compounded by immune‐complex deposition and concurrent endocrine disease, and supports consideration of regional antimicrobial delivery as a salvage strategy in refractory septic peritonitis when source control and systemic therapy alone prove insufficient.

## Graphical abstract

Chronic, recurrent septic peritonitis due to *Staphylococcus pseudintermedius* with Splendore‐Hoeppli reaction can be challenging to eradicate, particularly in the presence of diabetes mellitus, and the regional antimicrobial delivery may be considered as a salvage strategy when source control and systemic therapy alone are insufficient.

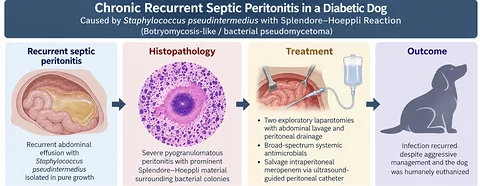

**Abbreviations**
aPTT: activated partial thromboplastin time
CT: computed tomography
DM: diabetes mellitus
EPS: extracellular polymeric substance
GMS: Grocott methenamine silver
IP: intraperitoneal
IV: intravenous
MBEC: minimum biofilm eradication concentration
MIC: minimum inhibitory concentration
PAS: periodic acid–Schiff
SH: Splendore–Hoeppli
SHP: Splendore–Hoeppli phenomenon
SIRS: systemic inflammatory response syndrome
SP: septic peritonitis
TXA: tranexamic acid

## 1 Background

Septic peritonitis (SP) is one of the most serious surgical emergencies encountered in small animal medicine, with reported mortality rates ranging between 20% and 85% depending on the underlying aetiology, timing of intervention and presence of concurrent disease (Ribeiro et al. 2024; Shipov et al. 2023; Kradangnga et al. 2024; Ylimartimo et al. 2022; Costello et al. 2004). SP most commonly arises from leakage of gastrointestinal contents into the peritoneal cavity following intestinal perforation, surgical dehiscence or neoplastic ulceration, but it may also develop secondary to biliary tract rupture, hepatobiliary disease, post‐surgical contamination, penetrating abdominal trauma, foreign body migration or hematogenous spread from a distant infectious focus. Once microbial contamination is established within the peritoneal cavity, a rapid inflammatory cascade ensues, characterized by neutrophilic infiltration, cytokine release, endothelial injury, vasodilation, capillary leakage and progressive third‐space fluid loss. In the absence of timely source control and aggressive supportive care, this process can rapidly progress to systemic inflammatory response syndrome (SIRS), septic shock and multiple organ dysfunction syndrome.

While most cases follow an acute, self‐limited course after surgical intervention, a clinically important subset evolves into a chronic or recurrent state. Chronic SP is characterized by fibrous peritoneal adhesions, persistent low‐grade inflammation and repeated relapses despite apparently appropriate medical and surgical treatment. This refractory pattern frequently reflects the formation of bacterial biofilms, in which microorganisms encase themselves within a sessile community embedded in a self‐produced matrix of polysaccharides, proteins and extracellular DNA, collectively termed the extracellular polymeric substance (EPS). Within this microenvironment, bacteria adopt an altered metabolic phenotype and are afforded substantial protection from both host immune defences and the bactericidal activity of antimicrobial agents (Singh et al. 2013; Hall and Mah 2017; Andrade et al. 2022; Pompilio et al. 2015). Biofilm‐mediated infections are notoriously difficult to eradicate, often persisting despite repeated surgical debridement and prolonged courses of systemic antimicrobials, thereby predisposing affected dogs to chronic, recurrent and progressive disease.

*Staphylococcus pseudintermedius* has emerged over the past two decades as an increasingly important cause of persistent infection in dogs. A coagulase‐positive commensal of the canine mucocutaneous flora, *S. pseudintermedius* can behave as an opportunistic pathogen when host defences are impaired and it possesses a diverse arsenal of virulence factors including adhesins, exotoxins, leucocidins and the capacity to elaborate robust biofilms on both biotic and abiotic surfaces. While most commonly implicated in pyoderma, otitis externa and surgical wound infections, the organism has been increasingly recognized as a cause of deeper invasive disease including discospondylitis, endocarditis, urinary tract infection and post‐surgical implant infection (Rosa et al. 2018; Van Duijkeren et al. 2011). When such infections become chronic, they frequently adopt a pyogranulomatous histopathologic pattern reflecting prolonged host–pathogen interaction and ineffective bacterial clearance (Pompilio et al. 2015; Rosa et al. 2018; Silva et al. 2022; Carroll et al. 2021).

A distinctive histopathologic feature occasionally accompanying chronic infections of this kind is the Splendore–Hoeppli (SH) phenomenon (Hussein 2008; Gopinath 2018). First described in the early 20th century in association with sporotrichosis and schistosomiasis, SH material appears microscopically as radiating, eosinophilic, club‐shaped or stellate structures surrounding microorganisms or other antigenic foci, and it is composed of antigen–antibody complexes, fibrin, immunoglobulins, tissue debris and degenerated inflammatory cells (Hussein 2008; Gopinath 2018). It is regarded as a localized immune‐mediated response in which deposition of immune complexes attempts to sequester antigenic material yet paradoxically forms a physical and biochemical barrier that limits phagocytosis and impairs antimicrobial penetration, thereby contributing to the chronicity of disease. While most commonly described in fungal infections and filamentous bacterial diseases such as actinomycosis, nocardiosis and botryomycosis, the phenomenon has also been documented in non‐filamentous bacterial infections, including *Staphylococcus* species, where it underlies the formation of bacterial pseudomycetomas (Hussein 2008; Gopinath 2018; Ecimovic et al. 2024; França et al. 2014; Read et al. 2005).

This report describes a case of recurrent, chronic SP in a dog caused by *S. pseudintermedius*, characterized histopathologically by severe pyogranulomatous inflammation and prominent SH material consistent with bacterial pseudomycetoma, and clinically complicated by poorly regulated diabetes mellitus (DM). To the authors' knowledge, this represents one of the few documented reports of *S. pseudintermedius*–associated bacterial pseudomycetoma involving the canine peritoneal cavity. The case is presented to highlight the diagnostic complexity of chronic intra‐abdominal staphylococcal infection, to illustrate the clinical features that should raise suspicion for biofilm‐associated persistence, and to describe an adjunctive regional antimicrobial strategy used as salvage therapy.

## 2 Case Presentation

A 6‐year‐old neutered male Miniature Pinscher weighing 5.6 kg was referred for evaluation of recurrent abdominal effusion, lethargy and reduced appetite. The dog had been diagnosed with DM 2 years prior to presentation and was being managed with porcine insulin zinc suspension (Vetsulin, Merck Animal Health). Historic glycaemic control had been suboptimal, with documented fluctuations between hyperglycaemia and episodic hypoglycaemia during intercurrent illnesses. No other chronic systemic conditions had been identified.

### 2.1 Medical and Surgical History

Four months before referral, the dog had undergone bilateral phacoemulsification for diabetes‐associated cataracts and had recovered without complications. Approximately one month following ocular surgery, he developed a right hip abscess from which aerobic culture yielded *Staphylococcus aureus* (2+) and *S. pseudintermedius* (1+). The abscess ruptured spontaneously and was managed with surgical debridement, primary wound closure, and a course of oral amoxicillin–clavulanate, with apparent complete clinical resolution. In retrospect, this prior staphylococcal infection may have served as a hematogenous reservoir or sentinel event predating the development of intra‐abdominal disease.

### 2.2 First Episode

The first episode of SP began in August 2025 and was characterized by acute onset of vomiting, abdominal distension, lethargy and inappetence. Initial diagnostic abdominocentesis yielded a serosanguinous exudate with marked neutrophilic inflammation. The dog was hospitalized overnight for supportive care; appetite returned and vomiting resolved, and he was discharged for outdog management pending advanced imaging. He returned several days later for abdominal ultrasonography, which revealed a moderate volume of echogenic peritoneal effusion, thickened hypoechoic mesentery and ill‐defined nodular foci (Figure 1). Repeat diagnostic abdominocentesis demonstrated marked neutrophilic inflammation with frequent intracellular cocci, consistent with septic exudative peritonitis. An exploratory laparotomy was performed on the dog, which revealed diffuse fibrinous peritonitis, marked serosanguinous effusion and pink proliferative tissue interspersed with white plaque‐like deposits across the omentum and mesentery. No intestinal perforation, biliary leakage, or discrete abscess was identified. The abdomen was lavaged extensively with warmed sterile saline and a closed‐suction Jackson–Pratt drain was placed.

**FIGURE 1:**
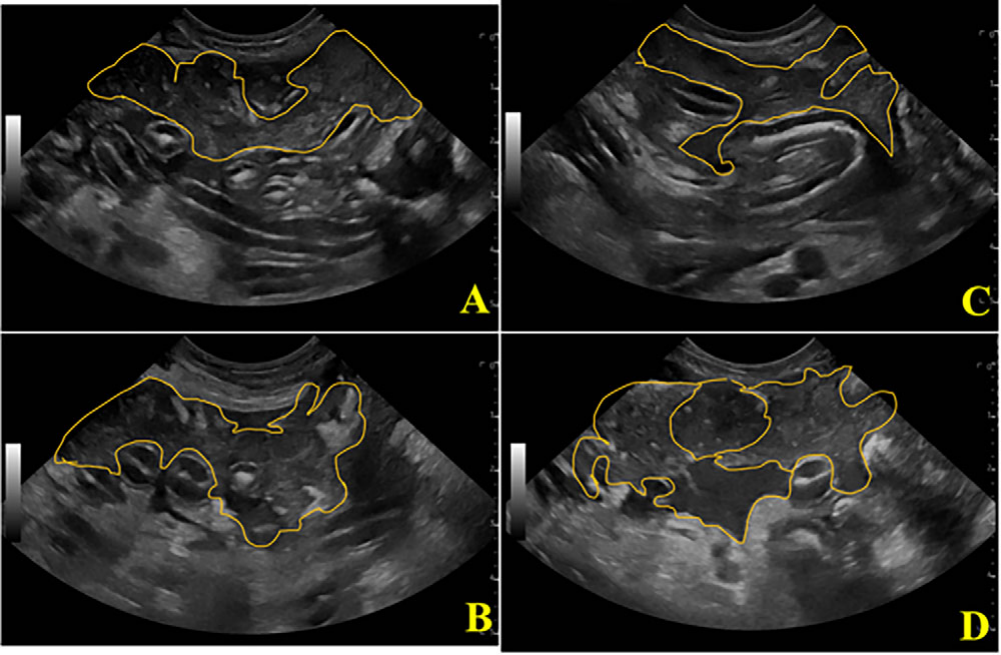
Abdominal ultrasound demonstrating diffuse mesenteric infiltration and peritoneal effusion (all outlined in yellow). (A) Irregular, heterogeneous hypoechoic mesenteric tissue surrounds multiple small‐intestinal loops with conspicuous loss of normal mesenteric fat echogenicity. Echogenic particulate effusion occupies the adjacent peritoneal space. (B) Similar regions of ill‐defined hypoechoic mesenteric expansion are seen enveloping vascular and intestinal structures, with suspended echogenic debris within the peritoneal fluid. (C) Additional loops of small intestine are bordered by thickened, infiltrated mesentery that creates broad, amorphous soft‐tissue zones. Echogenic peritoneal effusion persists in the dependent abdomen. (D) More caudal abdominal view demonstrating continued mesenteric infiltration and abundant echogenic fluid, outlining the extensive and generalized nature of the peritoneal involvement.

Aerobic culture of peritoneal fluid isolated *S. pseudintermedius* in pure growth, with documented susceptibility to enrofloxacin, amoxicillin–clavulanate, cefovecin, chloramphenicol, meropenem and rifampin and resistance to penicillin and amoxicillin alone. Histopathologic examination of omental biopsies demonstrated severe pyogranulomatous peritonitis with intralesional bacterial colonies surrounded by radiating, eosinophilic SH material, findings most consistent with bacterial pseudomycetoma.

Postoperatively, the dog was treated with intravenous ampicillin–sulbactam (30 mg/kg every 8 h) and enrofloxacin (10 mg/kg every 24 h). Recovery was complicated by the development of a haemoabdomen (packed cell volume 17%; 300 mL of haemorrhagic peritoneal effluent with a PCV of 26% recovered from the Jackson–Pratt drain), which was managed with transfusion of packed red blood cells (10 mL/kg) and tranexamic acid (TXA) administered every 6 h. Post‐transfusion anaemia remained stable. The peritoneal drain was removed at 48 h when daily output had declined to less than 2 mL/kg, and the dog was discharged on oral amoxicillin–clavulanate for 2 weeks. At a recheck examination 2 weeks following discharge, point‐of‐care ultrasonography demonstrated no residual peritoneal effusion; the dog was eating well without vomiting or lethargy, and the surgical incision had healed without complication.

### 2.3 Second Episode

Approximately 4 weeks after discharge from the first episode (6 weeks following initial diagnosis), the dog presented for renewed abdominal distension and reduced appetite. Point‐of‐care ultrasonography demonstrated findings consistent with recurrent peritonitis and re‐accumulation of peritoneal effusion. Repeat in‐hospital diagnostic abdominocentesis again revealed degenerate neutrophils containing intracellular cocci. A second exploratory laparotomy was performed. Intra‐abdominal findings closely mirrored those of the initial surgery, with diffuse peritoneal fibrin, proliferative omentum and white plaque‐like deposits along the mesentery; no perforation or discrete abscess was identified. The abdomen was again lavaged extensively and a closed‐suction Jackson–Pratt drain was placed.

Histopathology of omental plaques again demonstrated severe pyogranulomatous peritonitis with SH material and intralesional bacteria, consistent with chronic bacterial pseudomycetoma. Repeat culture of biopsy material isolated *S. pseudintermedius* with an antimicrobial susceptibility profile identical to that of the prior episode. Special stains (Ziehl–Neelsen, modified acid‐fast, periodic acid–Schiff [PAS] and Grocott methenamine silver [GMS]) were negative for *Actinomyces*, *Nocardia* and fungal organisms; fungal and rapid‐growing mycobacterial cultures were likewise negative.

Postoperative therapy again consisted of intravenous ampicillin‐sulbactam (30 mg/kg every 8 h) and enrofloxacin (10 mg/kg every 24 h). In‐house cytology of drain fluid 48 h after surgery demonstrated no bacteria, and the drain was removed. The dog was discharged once clinically stable on oral enrofloxacin and amoxicillin–clavulanate. Within 2 weeks, peritoneal effusion had resolved on imaging, and the surgical site had healed without complication. Amoxicillin–clavulanate therapy was continued beyond clinical resolution in an attempt to suppress any residual subclinical infection.

### 2.4 Third Episode

Four weeks after discharge from the second episode (approximately 10 weeks from initial diagnosis), the dog was presented to his primary veterinarian with lethargy, diarrhoea and swelling of the ventral neck and forelimbs. Laboratory testing at that visit revealed marked thrombocytopenia, mild anaemia and hyperglycaemia (Tables 1 and 2). He was referred to the speciality hospital the following day, where he remained lethargic and inappetent with persistent diarrhoea. An elevated body temperature was noted on admission. Physical examination demonstrated resolution of the forelimb swelling and only residual mild ventral cervical oedema. Point‐of‐care ultrasound demonstrated scant peritoneal effusion. Thoracic radiographs were within normal limits, and fructosamine concentration returned within the reference interval for adequate intermediate‐term glycaemic control. Diagnostic abdominocentesis was performed, and outdog management was elected pending peritoneal fluid cytology and culture. With concern for vasculitis, the dog was discharged on pentoxifylline in addition to ongoing amoxicillin–clavulanate.

**TABLE 1:** Complete blood count panels in chronological order of a 6‐year‐old Miniature Pinscher with chronic recurrent septic peritonitis starting the day of initial diagnosis.

Test	Day 1	Day 2	Day 42	Day 43	Day 69	Day 74	Day 76	Day 78	Day 82	Day 101	RI	Units
RBC	3.42 (L)	2.41 (L)	4.55 (L)	3.78 (L)	3.75 (L)	2.82 (L)	2.99 (L)	3.18 (L)	3.33 (L)	2.69 (L)	5.84–8.95	M/µL
HCT	23.7 (L)	17.0 (L)	31.5 (L)	27.7 (L)	28.1 (L)	20.3 (L)	21.3 (L)	22.7 (L)	23.0 (L)	18.2 (L)	41–60	%
HGB	7.6 (L)	5.5 (L)	10.5 (L)	8.9 (L)	8.6 (L)	6.3 (L)	6.8 (L)	7.2 (L)	*	6.2 (L)	14.6–21.7	g/dL
MCV	69	71	69.2	73	75	72	71	71	69	67.7	62–76	fL
MCHC	32.1 (L)	32.4	33.3	32.1 (L)	30.6 (L)	31.0 (L)	31.9 (L)	31.7 (L)	*	34.1	32.3–38	g/dL
RDW	15.3	15.4	15.4	13.2	14.1	13.8	14.0	14.0	14.3	17.6	10–19	%
ABS. RETIC	41.0	48.0	28.7	26.0	45.0	8.0 (L)	9.0 (L)	19.0 (L)	27	48.7	21–140	K/μuL
WBC	16.1	18.1 (H)	13.67	17.2 (H)	10.9	3.8 (L)	4.1 (L)	5.2 (L)	6.0	6.74	5.8–16.2	K/µL
NEUT	12.041 (H)	15.204 (H)	0.29 (L)	9.976 (H)	7.848	2.356 (L)	3.321	3.484	0.132 (L)	0.34 (L)	3.004–9.741	K/µL
BAND	0.644 (H)	0.724 (H)	Suspected	—	0.981 (H)	0.684 (H)	0.246 (H)	0.988 (H)	—	Suspected	0–0.17	K/µL
LYMPH	0.966 (L)	0.543 (L)	4.58 (H)	4.3 (H)	1.635	0.38 (L)	0.246 (L)	0.364 (L)	5.004 (H)	1.30	0.98–4.2	K/µL
MONO	0.483	0.543	8.50 (H)	2.408 (H)	0.218	0.076 (L)	0.164	0.26	0.643	4.80 (H)	0.145–0.736	K/µL
nRBC	4 (H)	5 (H)	—	7 (H)	3 (H)	—	—	—	—	—	0–2	Per 100 WBC
MPV	—	—	13.9 (H)	—	—	—	—	—	—	15.5 (H)	8.7–13.2	fL
PLT	231	100–adequate	116 (L)	143	53 (L)	26 (L)	31 (L)	32 (L)	50‐100 (L)	15 (L)	120–412	K/µL

*Note*: (H), high (above reference range); (L), low (below reference range); *, unable to determine the result due to lipaemia.

Abbreviations: ABS. RETIC, absolute reticulocyte count; BAND, band neutrophils; HCT, haematocrit; HGB, haemoglobin; LYMPH, lymphocytes; MCHC, mean corpuscular haemoglobin concentration; MCV, mean corpuscular volume; MONO, monocytes; MPV, mean platelet volume; NEUT, neutrophils; nRBC, nucleated red blood cells; PLT, platelets; RBC, red blood cells; RDW, red cell distribution width; RI, reference interval; WBC, white blood cells.

**TABLE 2:** Biochemistry panels in chronological order of a 6‐year‐old Miniature Pinscher with chronic recurrent septic peritonitis starting the day of initial diagnosis.

Test	Day 1	Day 2	Day 3	Day 42	Day 43	Day 69	Day 74	Day 75	Day 82	RI	Units
GLU	82	83	102	162	374 (H)	307 (H)	196 (H)	340 (H)	363 (H)	63–114	mg/dL
CREA	0.2 (L)	0.1 (L)	0.1 (L)	0.3 (L)	0.4 (L)	0.3 (L)	0.2 (L)	0.2 (L)	0.6	0.5–1.5	mg/dL
BUN	16	13	7 (L)	9	9	11	17	11	25	9–31	mg/dL
PHOS	4.6	4.1	3.1	4.5	4.6	4.2	3.3	4.0	4.4	2.5–6.1	mg/dL
CA	8.9	8.3 (L)	7.9 (L)	8.4	7.2 (L)	8.4	8.4	7.9 (L)	8.9	8.4–11.8	mg/dL
NA	158	164 (H)	167 (H)	155 (H)	154 (H)	154 (H)	151	153 (H)	153 (H)	142–152	mmol/L
K	4.9	3.8 (L)	5.2	5.0	4.8	4.5	4.4	4.5	4.0	4–5.4	mmol/L
CL	120 (H)	121 (H)	119	112	111	111	117	108	110	108–119	mmol/L
ALB	2.7	2.2 (L)	2.0 (L)	2.4 (L)	1.8 (L)	2.5 (L)	1.6 (L)	1.9 (L)	2.0 (L)	2.7–3.9	g/dL
GLOB	4.5 (H)	3.1	3.1	4.5 (H)	3.7	4.7 (H)	3.5	3.8	3.8	2.4–4	g/dL
ALT	43	38	31	18	31	< 10 (L)	10 (L)	17 (L)	50	18–121	U/L
ALP	208 (H)	202 (H)	147	205 (H)	136	244 (H)	221 (H)	312 (H)	346 (H)	5–160	U/L
GGT	2	0	0	0	0	0	1	0	4	0–13	U/L
T‐BILI	0.2	< 0.1	< 0.1	< 0.1	0.2	0.3	0.3	0.4	0.1	0–0.3	mg/dL
CHOL	253	192	176	236	188	297	459 (H)	302	402 (H)	131–345	mg/dL
TRIG	—	—	—	—	—	—	229 (H)	—	645 (H)	20–150	mg/dL
AMYL	595	385	458	452	327 (L)	585	618	331 (L)	437	337–1469	U/L
LIP	703 (H)	606 (H)	481 (H)	625 (H)	354 (H)	739 (H)	189	288 (H)	368 (H)	0–250	U/L
OSM	314	321	328	310	319	315	—	315	—	—	mmol/kg
CK	—	—	—	—	—	—	432 (H)	—	93	10–200	U/L

*Note*: (H), high (above reference range); (L), low (below reference range).

Abbreviations: ALB, albumin; ALP, alkaline phosphatase; ALT, alanine aminotransferase; AMYL, amylase; AST, aspartate aminotransferase; BUN, blood urea nitrogen; CA, calcium; CHOL, cholesterol; CK, creatine kinase; CL, chloride; CREA, creatinine; GGT, gamma‐glutamyl transferase; GLOB, globulin; GLU, glucose; K, potassium; LIP, lipase; NA, sodium; OSM, osmolality; PHOS, phosphorus; RI, reference interval; T‐BILI, total bilirubin; TRIG, triglyceride.

Cytologic evaluation of the peritoneal effusion again revealed numerous degenerate neutrophils with intracellular cocci, and culture again isolated *S. pseudintermedius* with an antimicrobial susceptibility profile identical to those of the omental tissue cultures from prior episodes.

The dog returned 2 days later following review of these diagnostic results. Hypoglycaemia and normotension were noted on admission. A coagulation panel confirmed persistent thrombocytopenia and revealed hyperfibrinogenaemia, a mildly prolonged activated partial thromboplastin time (aPTT), and a normal D‐dimer, raising concern for early or evolving disseminated intravascular coagulation. The dog was stabilized, and contrast‐enhanced computed tomography (CT) scan of the thorax and abdomen was performed, demonstrating severe peritoneal effusion, diffuse mesenteric enhancement and mild sternal lymphadenopathy (Figure 2). No gastrointestinal rupture, intra‐abdominal mass or discrete abscess was identified.

**FIGURE 2:**
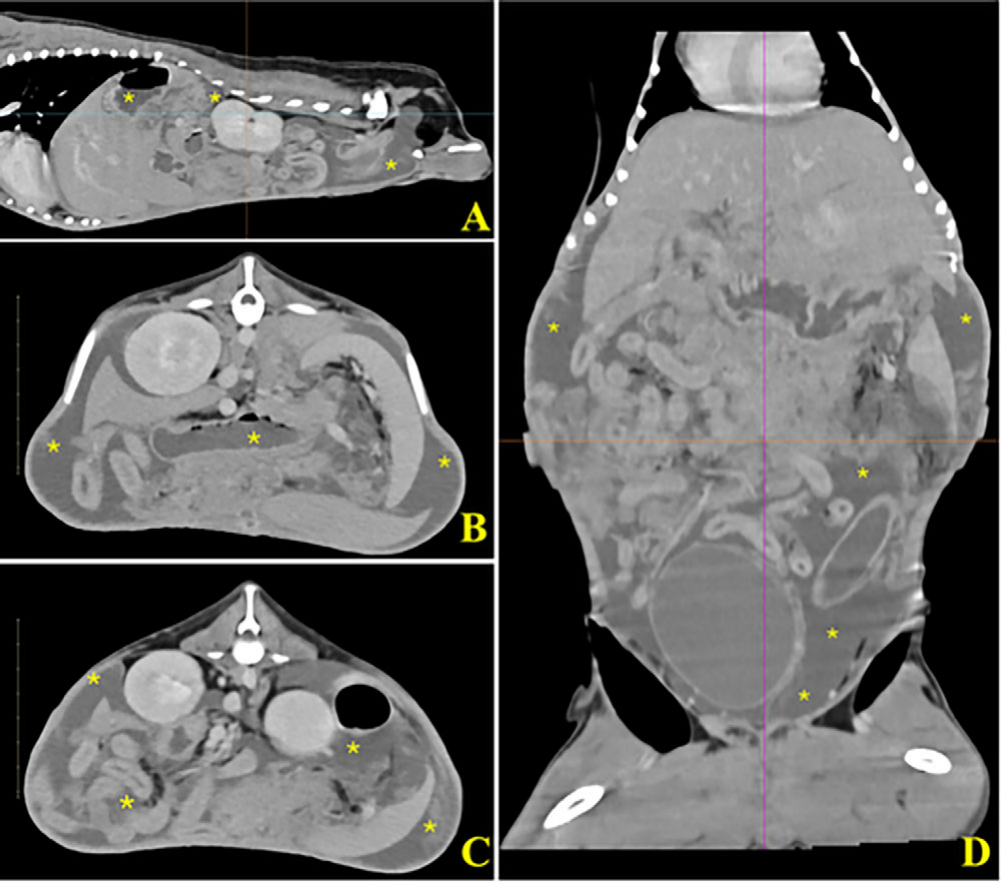
Contrast‐enhanced abdominal CT displayed in sagittal (A), axial (B, C) and dorsal (D) reconstructions. (A) Sagittal reconstruction shows a broad sheet of ill‐defined, mildly contrast‐enhancing soft tissue extending along the ventral abdomen and enveloping multiple intestinal segments (yellow stars indicate representative regions). (B) Axial image at a mid‐abdominal level highlights infiltration of the mesentery with amorphous soft tissue that surrounds mesenteric vessels and displaces adjacent bowel loops. (C) More caudal axial image demonstrates continued permeation of the mesenteric fat with similar soft‐tissue material, extending dorsally toward the spleen and producing mild organ displacement. (D) Dorsal reconstruction shows the full craniocaudal distribution of this infiltrative process throughout the peritoneal cavity, outlining extensive involvement of both the mesentery and peritoneal space without formation of a discrete mass. Yellow stars are all indicative of representative regions which correlated to the descriptions provided.

Given the recurrent and refractory nature of the infection and the absence of a surgically correctable source, the clinical team transitioned to non‐surgical medical management. An ultrasound‐guided sterile peritoneal catheter was placed, and intraperitoneal (IP) meropenem was initiated at 12 mg/kg every 8 h concurrent with intravenous meropenem at the same dose and frequency.

Supportive care included balanced isotonic crystalloids for volume replacement, intermittent norepinephrine infusion (0.1–0.2 µg/kg/min) for transient hypotension, and multimodal analgesia consisting of methadone, lidocaine and ketamine constant‐rate infusions. Enoxaparin (0.5 mg/kg subcutaneously every 12 h) was administered for sepsis‐associated coagulopathy. Insulin therapy was titrated daily under continuous glucose monitoring, with doses adjusted between 2 and 5 units twice daily to prevent hypoglycaemia. Antiemetic therapy with maropitant and ondansetron and nasogastric tube feeding were instituted to maintain enteral nutrition and minimize catabolism.

During hospitalization, the dog's clinical condition gradually improved. Abdominal distension subsided, and peritoneal drain output decreased to less than 2 mL/kg/day by the third day of therapy. Serial point‐of‐care ultrasonography demonstrated no residual effusion, and inflammatory markers progressively normalized (Table 1). After 3 days of combined intravenous and IP meropenem therapy, the dog was clinically bright, eating well and normothermic. In‐house cytology of drain fluid demonstrated no intracellular bacteria, and the peritoneal catheter was removed in preparation for discharge.

The dog was discharged on oral enrofloxacin (10 mg/kg once daily for 6 weeks), gabapentin for analgesia, and insulin at 3 units twice daily. The owner was instructed to return for ongoing glucose monitoring and serial haematology and imaging.

### 2.5 Fourth Episode

Approximately 4 weeks after discharge from the third episode (approximately 14 weeks from initial diagnosis), the dog returned for a scheduled blood glucose curve and was reported to have had soft stools and intermittent vomiting during the preceding week. Physical examination revealed abdominal distension with a positive fluid wave, tachypnoea, tachycardia and bounding pulses; body temperature and blood pressure were within normal limits. Point‐of‐care ultrasound demonstrated a large‐volume echogenic peritoneal effusion. Laboratory testing revealed mild hyperglycaemia, stable anaemia, marked thrombocytopenia and severe neutropenia. Therapeutic abdominocentesis was performed; cytology again revealed numerous degenerate neutrophils with suspected intracellular cocci, and repeat culture isolated *S. pseudintermedius* with a susceptibility profile similar to prior cultures.

The option of repeat hospitalization with ultrasound‐guided peritoneal catheter placement and another course of combined intravenous and IP antimicrobial therapy was discussed in detail with the owners. Given the suspected consumptive neutropenia and myeloid suppression, recurrent septic relapses despite escalating interventions, and the cumulative emotional and financial burden of ongoing care, the owners elected to forgo additional treatment, and humane euthanasia was pursued.

## 3 Investigations

A comprehensive diagnostic workup was undertaken at each clinical episode, with key hematologic, biochemical and inflammatory parameters summarized in Tables 1 and 2. Complete blood counts during the first episode demonstrated a moderate non‐regenerative anaemia (haematocrit 17%–21%) and a marked leucocytosis (white blood cell count 16.9 × 10^3^/µL) with a degenerative left shift. Platelet counts were within the reference interval on initial presentation but declined progressively across successive relapses, reaching 18 × 10^3^/µL during the third hospitalization and 15 × 10^3^/µL on the day of euthanasia. As the infection became chronic, the leukogram shifted toward severe neutropenia (nadir 0.34 × 10^3^/µL) with a relative lymphocytosis, findings consistent with suspected consumptive neutropenia and myeloid suppression secondary to chronic systemic inflammation, although marrow cytology was not performed to confirm this interpretation.

Serum biochemistry across all admissions, with the exception of the third episode, demonstrated persistent hyperglycaemia (316–506 mg/dL) attributable to poorly regulated DM. Hypalbuminaemia (1.6–2.3 g/dL) and hyperglobulinaemia (4.7–5.4 g/dL) were consistent features, reflecting chronic systemic inflammation, hepatic acute‐phase response and ongoing protein loss into the peritoneal cavity. The coagulation panel obtained during the third episode revealed hyperfibrinogenaemia, a slightly prolonged aPTT, and a normal D‐dimer in the presence of persistent thrombocytopenia, a constellation interpreted as concerning for early or evolving disseminated intravascular coagulation. C‐reactive protein concentrations were markedly elevated during septic exacerbations (range 59–83 mg/L) and declined toward reference range during periods of clinical improvement (Table 3). Mild hyperbilirubinemia, transient hypokalaemia and mild hyponatremia were also documented intermittently, while renal parameters remained within reference intervals throughout the disease course.

**TABLE 3:** Canine CRP values in a 6‐year‐old Miniature Pinscher with chronic recurrent septic peritonitis.

Test	Day 44	Day 74	Day 80	Day 88	RI	Units
Canine CRP	87.3 (H)	83.7 (H)	59.6 (H)	84.9 (H)	0.0–20.0	mg/L

*Note*: (H), high (above reference range).

Abbreviations: CRP, C‐reactive protein; RI, reference interval.

Abdominal ultrasonography at initial presentation revealed a large volume of echogenic peritoneal effusion, hypoechoic and thickened mesentery, and ill‐defined micronodular foci. Follow‐up imaging after the first surgery confirmed temporary resolution of effusion. Recurrence during the second episode was characterized by fluid reaccumulation, persistent mesenteric thickening and the development of focal adhesions. Contrast‐enhanced CT performed during the third hospitalization (Figure 2) demonstrated marked peritoneal effusion and diffuse mesenteric enhancement with mild sternal and cranial mediastinal lymphadenopathy. No gastrointestinal rupture, intra‐abdominal abscess or neoplastic mass was identified.

Cytologic evaluation of abdominocentesis samples from all episodes consistently revealed moderate to marked neutrophilic inflammation with frequent intracellular cocci and occasional macrophages containing intracytoplasmic bacterial clusters. No fungal hyphae or filamentous bacteria were identified on any cytologic preparation.

Aerobic culture of peritoneal fluid and omental tissue repeatedly yielded *S. pseudintermedius* in pure growth, while anaerobic and fungal cultures remained negative throughout. Antimicrobial susceptibility profiles were strikingly consistent across all submissions, demonstrating sensitivity to amoxicillin–clavulanate, enrofloxacin, cefovecin, chloramphenicol, meropenem and rifampin; resistance to penicillin and amoxicillin alone; and intermediate susceptibility to doxycycline (Table 1). The consistency of these profiles across multiple sampling events strongly suggested persistence of a single infecting strain rather than serial reinfection.

Histopathologic examination of omental biopsies obtained during both surgical episodes demonstrated severe, chronic pyogranulomatous peritonitis with intralesional bacterial colonies surrounded by radiating, eosinophilic SH material (Figure 3). Special stains, including Ziehl–Neelsen, modified acid‐fast, PAS and GMS, were uniformly negative for *Actinomyces*, *Nocardia* and fungal organisms. Aspergillus galactomannan and Blastomyces antigen enzyme immunoassays were both negative. Taken together, the cytologic, microbiologic and histopathologic findings were diagnostic for chronic bacterial pseudomycetoma associated with *S. pseudintermedius*.

**FIGURE 3:**
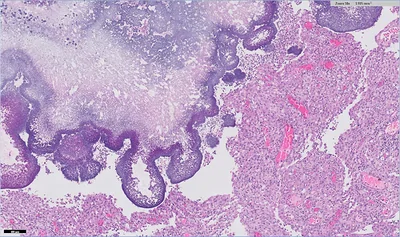
Histopathology of omentum using standard haematoxylin and eosin (H&E) stain. Interpretation: severe, pyogranulomatous peritonitis with intralesional Splendore–Hoeppli material and bacterial colonies. Abundant inflammation comprised of abundant neutrophils accompanied by foamy macrophages and perivascular to interstitial infiltrates of small mature lymphocytes and plasma cells. There are discrete pockets of densely packed neutrophils surrounding prominent radiating colonies of bacteria embedded within basophilic to magenta granular material. Inflammatory infiltrates are accompanied by organizing granulation tissue formation, congestion, mild haemorrhage or edema.

## 4 Differential Diagnosis

Differential diagnoses considered for the dog's chronic, recurrent peritonitis included actinomycosis, nocardiosis, mycobacterial infection, fungal peritonitis (including pythiosis and the systemic mycoses), sterile granulomatous peritonitis, foreign body–associated peritonitis, and neoplastic processes such as peritoneal carcinomatosis or mesothelioma. Actinomycotic and nocardial infections were initially considered foremost given the pyogranulomatous inflammatory pattern and SH reaction observed histologically; however, Ziehl–Neelsen and modified acid‐fast staining were negative, no filamentous organisms were identified on histopathology or cytology, and aerobic culture yielded only *S. pseudintermedius* in pure growth across multiple submissions. Fungal peritonitis attributable to Pythium insidiosum, Blastomyces dermatitidis, Histoplasma capsulatum, or Aspergillus species was excluded by negative fungal culture, negative antigen testing (Aspergillus galactomannan and Blastomyces antigen enzyme immunoassays), and the absence of fungal elements on PAS and GMS staining. Cytologic and histopathologic evaluation revealed no neoplastic cell population, no atypical mitotic activity and no reactive or malignant mesothelial proliferation to support a diagnosis of carcinomatosis or mesothelioma. Sterile granulomatous peritonitis, including idiopathic and foreign body–associated forms, was excluded by the repeated isolation of *S. pseudintermedius* in pure culture from both peritoneal fluid and omental tissue across all four clinical episodes. Collectively, these findings confirmed an etiologic diagnosis of chronic bacterial pyogranulomatous peritonitis with bacterial pseudomycetoma formation, secondary to *S. pseudintermedius* infection.

## 5 Treatment

Management of this case combined surgical exploration, aggressive antimicrobial therapy and intensive supportive care, with the therapeutic strategy evolving across episodes to address the increasingly refractory nature of the infection.

During the first episode, the dog underwent exploratory laparotomy that revealed diffuse fibrinous peritonitis, pink proliferative omental and mesenteric tissue and floating white plaque‐like deposits. No gastrointestinal perforation or visceral rupture was identified. The abdomen was lavaged extensively with warmed sterile saline, and a closed‐suction Jackson–Pratt drain was placed to facilitate ongoing evacuation of contaminated effusion. Postoperative antimicrobial therapy consisted of intravenous amoxicillin–clavulanate (20 mg/kg every 8 h), transitioned to oral administration (20 mg/kg every 12 h) for 2 weeks following clinical stabilization. Recovery was complicated by the development of a haemoabdomen, with a post‐surgical haematocrit of 17% and 300 mL of haemorrhagic effusion (PCV 26%) recovered from the abdominal drain. This was managed with transfusion of packed red blood cells (10 mL/kg) and TXA administered every 6 h, with subsequent stabilization of the haematocrit. The drain was removed once daily output decreased to less than 2 mL/kg/d.

Following recurrence in September, a second exploratory laparotomy was performed. Intra‐abdominal findings closely paralleled those of the first surgery, with diffuse serosal fibrin deposition, thickened omentum and white nodular plaques consistent with granulomatous inflammation. Extensive abdominal lavage and debridement of grossly abnormal omental tissue were repeated, and a second Jackson–Pratt drain was placed. Postoperative antimicrobial therapy was broadened to intravenous ampicillin–sulbactam (30 mg/kg every 8 h) and enrofloxacin (10 mg/kg every 24 h). The dog stabilized over several days and was discharged on oral enrofloxacin (10 mg/kg every 24 h) and amoxicillin–clavulanate acid (20 mg/kg every 12 h), with the latter continued beyond clinical resolution in an attempt to suppress residual subclinical infection.

The third episode was managed primarily by non‐surgical means in the absence of an identifiable surgically correctable source and given the substantial cumulative risk associated with further laparotomy. Thus, an ultrasound‐guided peritoneal catheter (a multi‐fenestrated sterile 12‐gauge MILA catheter) was placed into the right caudoventral abdomen using full aseptic technique and local anaesthesia. The abdomen was then decompressed by drainage of free peritoneal effusion prior to the first IP dose of regional meropenem. The selection of meropenem was supported by documented susceptibility of the isolate to multiple β‐lactams, the superior stability of carbapenems against β‐lactamase degradation, and the favourable peritoneal and interstitial fluid penetration demonstrated for this agent in canine pharmacokinetic studies (Mzyk et al. 2026; Bidgood and Papich 2002). IP administration was selected with the intent of maximizing local drug exposure within dense pyogranulomatous and fibrinous tissue matrices, where systemic antimicrobial penetration is typically poor, with the additional goal of targeting biofilm‐associated bacterial subpopulations relatively inaccessible to systemic therapy (Vlaar et al. 2013; Campbell 2025). Antimicrobial selection and route were reviewed in accordance with antimicrobial stewardship principles and were guided by repeated culture and susceptibility data from prior episodes (Tuon et al. 2023).

Meropenem was reconstituted in 0.9% sodium chloride to a final concentration of 20 mg/mL and administered intraperitoneally at 12 mg/kg every 8 h (3.6 mL or 72 mg). The 3.6 mL was diluted in an additional 20 mL of warmed 0.9% sodium chloride as a carrier vehicle to facilitate IP distribution. Following infusion, the catheter was clamped and a dwell time of 1 h was maintained before the catheter was reopened to gravity drainage; the abdomen was not flushed after dosing. The patient was repositioned every 30 min during the dwell to promote distribution within the peritoneal cavity. Intravenous meropenem was administered at the same dose and frequency, with each intravenous dose offset from the IP dose by approximately 1 h to maintain sustained antimicrobial exposure. The catheter site was inspected at least every 12 h for evidence of leakage, infection or dislodgement, and the patient was monitored for abdominal pain, respiratory compromise and signs of systemic toxicity at each dosing interval. No adverse effects attributable to IP therapy were observed.

The selection of meropenem was supported by documented susceptibility of the isolate to multiple β‐lactams, the superior stability of carbapenems against β‐lactamase degradation, and the favourable peritoneal and interstitial fluid penetration demonstrated for this agent in canine pharmacokinetic studies (Mzyk et al. 2026; Bidgood and Papich 2002). IP administration was selected with the intent of maximizing local drug exposure within dense pyogranulomatous and fibrinous tissue matrices, where systemic antimicrobial penetration is typically poor, with the additional goal of targeting biofilm‐associated bacterial subpopulations relatively inaccessible to systemic therapy (Vlaar et al. 2013; Campbell 2025). Antimicrobial selection and route were reviewed in accordance with antimicrobial stewardship principles and were guided by repeated culture and susceptibility data from prior episodes (Tuon et al. 2023).

Supportive care consisted of isotonic crystalloid fluid therapy for resuscitation and maintenance, short‐term norepinephrine infusion (0.1–0.2 µg/kg/min) for episodes of transient hypotension, and multimodal analgesia with methadone, lidocaine and ketamine constant‐rate infusions. Enoxaparin (0.5 mg/kg SC every 12 h) was administered prophylactically for sepsis‐associated coagulopathy. Antiemetic therapy with maropitant and ondansetron, together with nasogastric tube feeding, was employed to maintain enteral nutritional support and minimize catabolism.

Management of DM required frequent insulin adjustments guided by continuous glucose monitoring. Doses of porcine insulin zinc suspension varied between 2 and 11 units twice daily depending on appetite, infection severity and glycaemic fluctuations. Hypoglycaemic episodes (blood glucose < 70 mg/dL) during hospitalization were addressed with intravenous dextrose supplementation and temporary reduction or withholding of insulin. Once clinical stability was achieved, maintenance insulin therapy was instituted at 3 units twice daily.

The dog's hematologic abnormalities were monitored conservatively with serial complete blood counts and meticulous handling to avoid iatrogenic haemorrhage, given the progressive thrombocytopenia. A single packed red blood cell transfusion was administered after the first surgery, after which subsequent anaemia remained stable without further transfusion requirements.

Empirical fluconazole (10 mg/kg PO every 24 h) was briefly added early in the third hospitalization on the basis of clinical concern for occult fungal or mixed infection but was discontinued when fungal antigen testing returned negative.

After 3 days of combined intravenous and IP meropenem therapy, peritoneal drainage decreased to less than 2 mL/kg/d, and serial point‐of‐care ultrasonography confirmed complete resolution of abdominal effusion. The peritoneal catheter was removed, and the dog was transitioned to oral enrofloxacin (10 mg/kg every 24 h) for an additional 6 weeks as continuation therapy.

## 6 Outcome and Follow‐Up

Following discharge from the third episode, serial blood glucose curves performed at 5 and 12 days post‐discharge demonstrated persistent diabetic dysregulation while the patient was maintained on 3 and subsequently 4 units of insulin twice daily. At the 4‐week follow‐up after discharge from the third episode, the patient presented with recurrent peritoneal effusion accompanied by clinical, hematologic and imaging findings consistent with severe systemic inflammation, evolving coagulopathy, immune dysregulation and progressive hematopoietic suppression associated with chronic sepsis.

Despite intervals of clinical improvement following each prior surgical and medical intervention, the long‐term prognosis was considered guarded to poor. The suspected biofilm‐associated nature of the *S. pseudintermedius* infection and the apparent immunologic organization of the inflammatory response, in combination with the immunomodulatory effects of poorly regulated DM, posed ongoing and cumulative risk for further relapse and progressive systemic deterioration. After detailed discussion of further treatment options and their attendant risks and likely outcomes, the owners elected humane euthanasia. Post‐mortem examination was not authorized, which precluded definitive assessment of the extent of intra‐abdominal disease, exclusion of occult sources and characterization of bone marrow cellularity.

## 7 Discussion

This case illustrates an uncommon and clinically instructive manifestation of chronic recurrent SP in a dog, in which infection with *S. pseudintermedius* produced severe pyogranulomatous peritonitis associated with prominent SH reaction and bacterial pseudomycetoma formation. Recurrent bacterial peritonitis of this character is rarely reported in the veterinary literature and poses substantial diagnostic and therapeutic challenges. Unlike typical secondary SP, in which contamination arises from a discrete event such as gastrointestinal perforation, biliary leakage or organ rupture (Shipov et al. 2023; McGrofty and Doust 2004; Barfield et al. 2016; Culp and Holt 2010), the absence of an identifiable surgical source in this patient suggested a self‐sustaining, compartmentalized intra‐abdominal infection perpetuated by biofilm formation and immune‐complex deposition rather than ongoing contamination from a luminal source.

The pathogenesis of the Splendore–Hoeppli phenomenon (SHP) has historically been most strongly associated with actinomycotic, nocardial and fungal infections (Singh et al. 2013; Read et al. 2005; Padilla‐Desgarennes et al. 2012; Sivacolundhu et al. 2001; Lee et al. 2025), but it has also been documented in association with *Staphylococcus* species (Hussein 2008), in which chronic antigenic stimulation results in local deposition of immune complexes, fibrin, immunoglobulins and necrotic cellular debris (Kradangnga et al. 2024; Hussein 2008) around bacterial microcolonies. These eosinophilic, radiating structures (Kradangnga et al. 2024; Hussein 2008) are thought to function as physical and biochemical barriers to phagocytosis and antimicrobial penetration (Ylimartimo et al. 2022), promoting bacterial survival within pyogranulomatous tissue (Hussein 2008; Lee et al. 2025). The repeated identification of SH material on histopathology in this case supported progression toward a chronic, pyogranulomatous, immunologically organized infectious state (Hussein 2008; Padilla‐Desgarennes et al. 2012; Lee et al. 2025), in which encapsulation of bacterial niches by granulomatous tissue (Mzyk et al. 2026) is likely to impair the delivery of bactericidal antimicrobial concentrations from systemic circulation and from IP lavage, providing the rationale for alternative routes of antimicrobial delivery (Goggs et al. 2022). The histologic appearance showcases bacterial microcolonies surrounded by radiating eosinophilic material in the absence of identifiable filamentous organisms. This is most consistent with bacterial pseudomycetoma, also termed botryomycosis, a chronic suppurative infection most often caused by *Staphylococcus* species but also reported with *Pseudomonas*, *Streptococcus* and *Escherichia coli*.

*S. pseudintermedius* is recognized as a clinically significant opportunistic pathogen in dogs and has been increasingly implicated in chronic infections believed to involve biofilm formation. Within biofilms, bacterial populations exist in a sessile, phenotypically altered state demonstrating up to a 1000‐fold increase in tolerance to antimicrobial agents compared with their planktonic counterparts (Pompilio et al. 2015; Silva et al. 2022). The recurrent peritoneal effusion, persistent inflammatory adhesions and gross omental appearance described in this dog were consistent with such a process, although biofilm involvement could not be directly confirmed in the absence of molecular typing, scanning electron microscopy or biofilm‐specific in vitro assays. The identical antimicrobial susceptibility profile observed across multiple culture submissions is suggestive of, but not diagnostic for, persistence of the original infecting strain rather than serial reinfection. The patient's prior history of mixed staphylococcal hip abscess and bilateral phacoemulsification raises the speculative possibility of perioperative inoculation or hematogenous seeding from a cutaneous reservoir as the originating event; however, in the absence of contemporaneous blood cultures, documentation of bacteremia, or molecular comparison of isolates, this association cannot be substantiated and is offered only as a hypothesis.

The decision to administer meropenem both systemically and via the IP route was guided by culture and susceptibility data and by pharmacologic considerations specific to the chronic, compartmentalized nature of this infection. Although the isolate was susceptible to several β‐lactam agents, carbapenems offer superior stability against β‐lactamase degradation and broad‐spectrum, time‐dependent bactericidal activity with favourable penetration into inflamed peritoneal surfaces (Mzyk et al. 2026; Bidgood and Papich 2002). Canine pharmacokinetic studies have demonstrated that meropenem achieves high plasma and interstitial fluid concentrations following parenteral administration, supporting its clinical use for deep‐seated or compartmentalized infections (Bidgood and Papich 2002). Experimental data from both human and animal peritonitis models indicate that IP meropenem administration produces local concentrations exceeding both the minimum inhibitory concentration (MIC) and the minimum biofilm eradication concentration (MBEC) for *Staphylococcus* species without producing systemic toxicity (Vlaar et al. 2013; Campbell 2025). In this case, IP therapy was specifically intended to target residual bacterial foci embedded within granulomatous and fibrinous matrices, where intravenous antimicrobials alone were unlikely to achieve sustained bactericidal concentrations. The combined systemic and IP strategy thus represented a pharmacodynamically rational and stewardship‐conscious approach, maximizing local efficacy while limiting cumulative duration of systemic carbapenem exposure and the attendant risk of resistance selection (Vlaar et al. 2013; Campbell 2025; Tuon et al. 2023). Importantly, the use of carbapenems in veterinary patients should remain restricted to documented multidrug‐sensitive infections in which suitable alternatives are unavailable, and should always be prescribed under formal antimicrobial stewardship oversight.

DM further complicated both the pathophysiology and clinical management of this infection. Chronic hyperglycaemia is well recognized to impair neutrophil chemotaxis, phagocytosis and oxidative burst function, while also promoting microvascular dysfunction, accumulation of advanced glycation end‐products and delayed wound healing (Nelson and Reusch 2014). These mechanisms collectively contribute to impaired bacterial clearance and likely played a role in the dog's prolonged recovery course and pronounced susceptibility to relapse. In parallel, the systemic inflammatory burden of sepsis interferes with insulin signalling and induces a state of relative insulin resistance, generating a bidirectional cycle in which infection destabilizes glycaemic control and dysglycaemia in turn worsens immune dysfunction (Wei et al. 2022; Kaneki 2017). Effective and stable glycaemic control was therefore central to any meaningful attempt at infection resolution. The episodic hypoglycaemia documented during periods of acute illness in this patient illustrates the additional challenge of safely titrating insulin therapy in septic diabetic patients with fluctuating appetite, fluid balance and metabolic demand.

The principal limitations of this report stem from its description of a single clinical case, which inherently limits generalizability and precludes establishing direct causation between specific interventions and observed outcomes. Nevertheless, given the rarity of chronic recurrent SP attributable to *S. pseudintermedius* with concurrent SH reaction, individual case documentation remains valuable for advancing clinical recognition and refining pathophysiologic understanding. Molecular typing and biofilm‐specific assays, such as crystal violet quantification or confocal microscopy with live/dead staining, were not performed; however, the organism was repeatedly cultured in pure culture with consistent antimicrobial profiles across multiple sampling sites and time points, strongly supporting persistence of a single strain and biofilm‐associated infection as the most plausible mechanism. Quantitative pharmacokinetic and pharmacodynamic data for IP meropenem in dogs are not currently available, but dosing in this case was derived from established pharmacologic principles and prior experimental data from veterinary and human peritoneal infection models demonstrating safety and efficacy of the IP route. Histopathologic interpretation of the SH material was based on conventional histochemical staining, which reliably identifies this reaction pattern in both bacterial and fungal disease; immunohistochemical or molecular confirmation would be unlikely to alter diagnostic interpretation given the supportive culture data. A post‐mortem examination, which might have further characterized the extent of disease and excluded occult sources of infection, was not authorized. Finally, although long‐term follow‐up was ultimately limited by relapse and humane euthanasia, multiple clinical, imaging and laboratory data points across episodes documented repeated transient resolution of disease following both surgical and medical interventions, with a comparable time course of source control achieved by combined intravenous and IP antimicrobial therapy as by exploratory laparotomy with extensive lavage.

In summary, this case showcases the importance of correlating cytologic, microbiologic and histopathologic findings in any case of refractory peritonitis. The recognition of SH structures on histopathology should prompt consideration of bacterial pseudomycetoma even in the absence of identifiable filamentous bacteria. In chronic infections caused by *S. pseudintermedius*, suspected biofilm formation and immune‐complex deposition may render conventional surgical and systemic antimicrobial therapy inadequate, and adjunctive regional antimicrobial delivery, such as IP carbapenem administration, may represent a reasonable salvage option when infection persists despite appropriate first‐line interventions. Such strategies should be reserved for culture‐confirmed, multidrug‐susceptible infections under strict antimicrobial stewardship oversight. Clinicians managing such cases should additionally remain alert to the development of secondary systemic complications, including anaemia of chronic inflammation, sepsis‐associated coagulopathy and disseminated intravascular coagulation, and hematopoietic suppression associated with chronic sepsis, all of which may emerge insidiously and significantly influence the long‐term prognosis.

## 8 Learning Points

- Chronic recurrent SP without an identifiable surgical source should raise suspicion for biofilm‐associated bacterial infection. The histopathologic finding of SH material surrounding bacterial colonies, even without filamentous organisms, should prompt consideration of bacterial pseudomycetoma.
- When refractory SP persists despite source control and culture‐guided systemic therapy, regional antimicrobial delivery via an ultrasound‐guided peritoneal catheter, particularly with a carbapenem such as meropenem, may be considered as a stewardship‐bound salvage option for culture‐confirmed, susceptible infections.
- Poorly regulated DM and chronic sepsis form a bidirectional cycle of impaired immune function and dysglycaemia; vigilance for secondary complications, including coagulopathy and hematopoietic suppression, is essential to long‐term management.

## Author Contributions

**Diamond Taylor**: conceptualization (lead); investigation (lead); data curation (lead); writing – original draft (equal); writing – review and editing (equal). **Kyle L. Granger**: supervision (equal); project administration (lead); writing – original draft (equal); writing – review and editing (equal). **Anne Cohen**: supervision (equal); writing – review and editing (equal). All authors reviewed and approved the final version of the manuscript and agree to be accountable for their respective contributions.

## Funding

The authors have nothing to report.

## Ethics Statement

The work described in this manuscript involved the use of non‐experimental (owned or unowned) animals. Established internationally recognized high standards (‘best practice’) of veterinary clinical care for the individual patient were always followed. Ethical approval from a committee was therefore not specifically required for publication.

## Conflicts of Interest

The authors declare no conflicts of interest.

## Data Availability

Data sharing not applicable to this article as no datasets were generated or analysed during the current study.
